# Sepsis-Induced Gut Dysbiosis Mediates the Susceptibility to Sepsis-Associated Encephalopathy in Mice

**DOI:** 10.1128/msystems.01399-21

**Published:** 2022-06-01

**Authors:** Heng Fang, Yirong Wang, Jia Deng, Huidan Zhang, Qingrui Wu, Linling He, Jing Xu, Xin Shao, Xin Ouyang, Zhimei He, Qiuping Zhou, Huifang Wang, Yiyu Deng, Chunbo Chen

**Affiliations:** a The Second School of Clinical Medicine, Southern Medical University, Guangzhou, China; b Department of Critical Care Medicine, Guangdong Provincial People's Hospital, Guangdong Academy of Medical Sciences, Guangzhou, China; c Department of Intensive Care Unit of Cardiac Surgery, Guangdong Cardiovascular Institute, Guangdong Provincial People’s Hospital, Guangdong Academy of Medical Sciences, Guangzhou, China; d Clinical Research Center, Maoming People’s Hospital, Maoming, Guangdong, China; e Department of Critical Care Medicine, The Second Hospital of Dalian Medical University, Dalian, China; University of California San Diego

**Keywords:** gut microbiota, sepsis-associated encephalopathy, indole-3-propionic acid, microglia

## Abstract

Sepsis-associated encephalopathy (SAE) is common in septic patients and is associated with adverse outcomes. The gut microbiota has been recognized as a key mediator of neurological disease development. However, the exact role of the gut microbiota in regulating SAE remains elusive. Here, we investigated the role of the gut microbiota in SAE and its underlying mechanisms. Cecal ligation and puncture (CLP) was conducted to induce sepsis in mice. Neurological scores were recorded to distinguish SAE-resistant (SER) (score of >6 at 36 h postoperatively) from SAE-susceptible (SES) (score of ≤6 at 36 h postoperatively) mice. 16S rRNA gene sequencing and metabolomics analyses were used to characterize the gut microbiota in the two groups. Fecal microbiota transplantation was performed to validate the role of the gut microbiota in SAE progression. The gut microbiota was more severely disrupted in SES mice than in SER mice after sepsis modeling. Interestingly, mice receiving postoperative feces from SES mice exhibited more severe cortical inflammation than mice receiving feces from SER mice. Indole-3-propionic acid (IPA), a neuroprotective molecule, was more enriched in feces from SER mice than in feces from SES mice. IPA alleviated CLP-induced anxiety and spatial memory impairment in septic mice. Moreover, IPA markedly inhibited NLRP3 inflammasome activation and interleukin-1β (IL-1β) secretion in lipopolysaccharide-stimulated microglia. These responses were attenuated after antagonizing the aryl hydrocarbon receptor. Our study indicates that the variability in sepsis-induced gut dysbiosis mediates the differential susceptibility to SAE in CLP-induced experimental sepsis mice, and microbially derived IPA is possibly involved in SAE development as a neuroprotective compound.

**IMPORTANCE** The bidirectional interactions between the gut microbiota and sepsis-associated encephalopathy (SAE) are not well characterized. We found that the gut microbiota was more severely disturbed in SAE-susceptible (SES) mice than in SAE-resistant (SER) mice after sepsis modeling. Mice gavaged with postoperative feces from SES mice exhibited more severe neuroinflammation than mice gavaged with feces from SER mice. The gut microbiota from SER mice enriched a neuroprotective metabolite, IPA, which appeared to protect mice from SAE. The potential underlying mechanism of the protective effect of IPA may be mediated via the inhibition of NLRP3 inflammasome activation and IL-1β secretion in microglia. These anti-inflammatory effects of IPA may be regulated by aryl hydrocarbon receptors. These results enhance our understanding of the role of the intestinal microbiota in sepsis. In particular, gut microbiota-derived IPA may serve as a potential therapeutic agent to prevent neuroinflammation in SAE.

## INTRODUCTION

Sepsis is a dysregulated host response to microbial infection that leads to organ damage and is one of the leading causes of death in critically ill patients (1). Every year, millions of people worldwide are impacted by sepsis, and between one-third and one-sixth of these people succumb to the disease (1). Patients with sepsis often develop brain dysfunction (2–4). Sepsis-associated encephalopathy (SAE) is a risk factor for adverse outcomes (5). In addition, many sepsis survivors continue to suffer from long-term deficits, including cognitive impairment, psychiatric disorders, and impaired quality of life (6). There is a lack of treatment modalities that can reduce the burden of this life-threatening brain dysfunction (7). Therefore, the elucidation of the pathogenetic mechanisms of SAE and development of effective prevention and treatment strategies are key priorities.

There is considerable interindividual variability with respect to SAE susceptibility. For example, septic patients with acute kidney injury and common metabolic disturbances are more susceptible to SAE (8). However, the underlying mechanisms of differential susceptibility to SAE in patients have yet to be elucidated. Previous studies have indicated that susceptibility to organ injury, especially in the brain, could be modulated by the intestinal microbiota (9–11). In addition, microbial metabolites have been increasingly recognized as key mediators of the functional effects of gut-brain communication (11). The role of the microbiota-gut-brain axis in the pathophysiology of neuropsychiatric and neurological disorders is gaining increasing attention (11–13). However, the bidirectional interactions between the gut microbiota and SAE are not well characterized.

Patients with sepsis exhibit severe disruptions in the gut microbiota profile for various reasons, such as the use of broad-spectrum antibiotics (14, 15). Furthermore, gut dysbiosis adversely affects the prognosis of critically ill patients (16). Moreover, fecal microbiota transplantation (FMT) and probiotic treatment have been shown to improve the survival rate of patients with sepsis and alleviate the symptoms of encephalopathy (17, 18). Microbial metabolites are believed to exhibit protective effects that can suppress neuroinflammation in some neurological diseases (11). Based on these findings, we hypothesized that the susceptibility of septic patients to SAE is attributable to sepsis-induced gut dysbiosis accompanied by a decrease in certain beneficial metabolites. In the present study, we aimed to investigate the role of the gut microbiota in modulating susceptibility to SAE and to identify the protective metabolites involved. Our findings may provide evidence for a novel therapeutic strategy for the prevention of this disease.

## RESULTS

### Characterization of SAE-resistant and SAE-susceptible mice.

**FIG 1:**
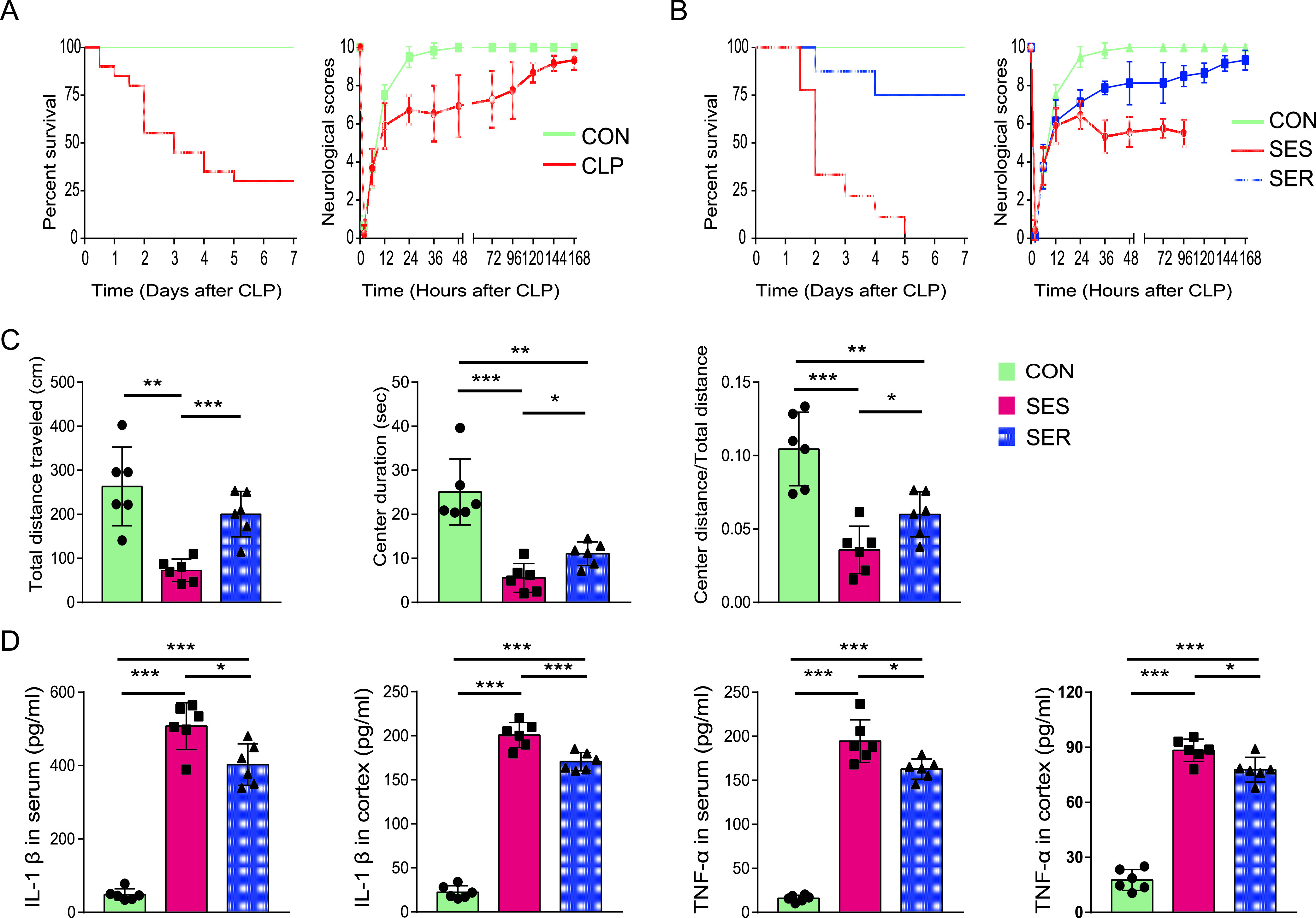
Characterization of SAE-resistant and SAE-susceptible mice. Mice with a neurological score of ≤6 at 36 h were defined as SAE susceptible (SES), and mice with a score of >6 were defined as SAE resistant (SER). (A) Survival rates and neurological scores of all mice within 7 days after operation (*n* = 20). (B) Survival rates and neurological scores of the SES, SER, and CON groups (*n* = 8 to 9). (C) The total distance traveled, center duration, and the ratio of center distance to total distance were collected and analyzed (*n* = 6). (D) Levels of proinflammatory cytokines in the serum and cortex (*n* = 6). *, *P *< 0.05; **, *P *< 0.01; ***, *P *< 0.001.

The neurological scores and survival rates of mice at different time points after surgery were recorded. In the mice that underwent operation, the scores of the neurological assessment were lowest at 2 h; this was followed by a gradual recovery of the neurological responses between 2 and 24 h. The neural reflexes of sham-operated mice (CON) returned to normal within 24 to 48 h. However, the scores of the cecal ligation and puncture (CLP)-operated mice were decreased at 36 h, which was followed by a gradual increase from 48 h to 7 days (Fig. 1A). In addition, the standard deviation of scores in the CLP group showed an obvious increase at 36 h (Fig. 1A), which indicates considerable interindividual variability in neural reflexes of septic mice at 36 h. Survival analysis showed high mortality in CLP-operated mice within 48 h (Fig. 1A). Therefore, mice with a neurological score of ≤6 at 36 h were defined as SAE susceptible (SES), while mice with a score >6 were defined as SAE resistant (SER). There was no significant difference in the neurological scores between the SES and SER groups in the first 24 h; however, the scores in the SES group were lower than those in the SER group at 36 h and thereafter (Fig. 1B). In addition, the survival of SER mice was better than that of SES mice (Fig. 1B).

We further compared the extent of behavioral changes among these three groups. Compared to the control group, mice in the CLP group performed worse in the open field test (Fig. 1C). The total distance traveled, duration spent in the center, and ratio of center distance to total distance of mice in the SES group were significantly lower than those in the SER group at 36 h. These findings suggest that the SES mice showed greater anxiety and worse mobility (Fig. 1C). In addition, we evaluated systemic inflammation and neuroinflammation in the mice that underwent an operation. Interleukin-β (IL-β) and tumor necrosis factor alpha (TNF-α) levels in both the serum and cortex of CLP-operated mice were significantly elevated compared to those in the sham-operated mice. In both the serum and cortex, SES mice showed higher levels of IL-β and TNF-α than SER mice, which indicates more pronounced systemic inflammation and neuroinflammation in SES mice (Fig. 1D).

### CLP-induced variations in the composition of the gut microbiota in SES and SER mice.

**FIG 2:**
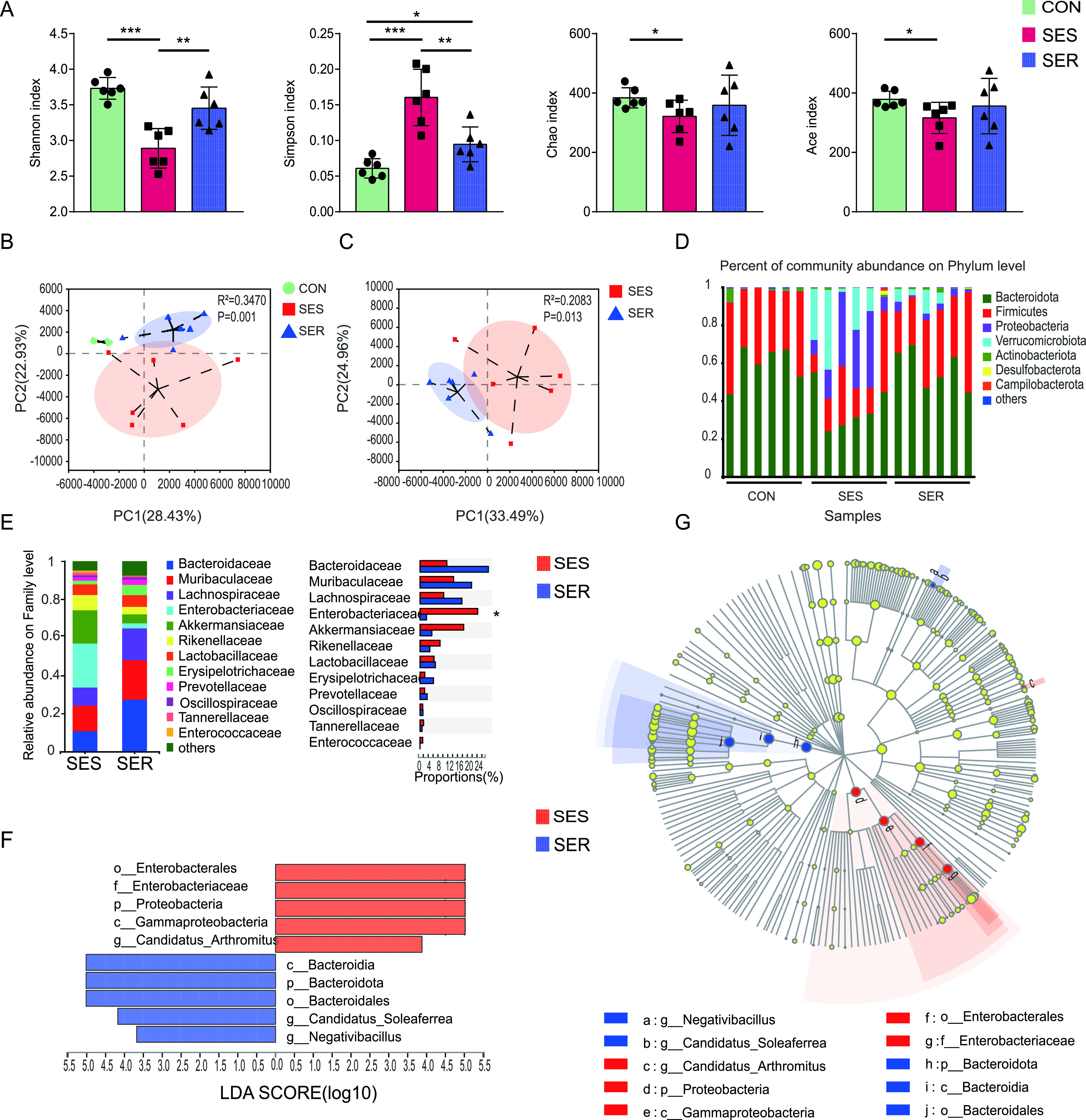
Variations in the composition of the gut microbiota caused by sepsis in mice. Fecal samples of mice were collected on day 2 after CLP. (A) Composition of α-diversity (as assessed by the Shannon, Simpson, Chao, and Ace indices) of feces. (B, C) Scatterplots of PCA for gut microbiota composition showing the β-diversity of feces. (D) Relative abundance of the gut microbiota at the phylum level. (E) Relative abundance of the gut microbiota at the family level. (F, G) LEfSe analysis of the gut microbiota. (F) Linear discriminant analysis (LDA) scores. (G) Taxonomic cladogram obtained from LEfSe analysis of 16S sequences. *n* = 6. *, *P *< 0.05; **, *P *< 0.01; ***, *P *< 0.001.

To explore whether the susceptibility of mice to SAE is related to preoperative differences in the microbiota or to sepsis-induced differences in the microbiota, we analyzed the gut microbiota composition of mice before and after CLP. The Shannon, Simpson, Ace, and Chao indices were calculated to evaluate the bacterial α-diversity in various groups, and principal-component analysis (PCA) was used to assess β-diversity. There was no significant difference between SES and SER mice with respect to α- or β-diversity before CLP (see Fig. S1A to C in the supplemental material), suggesting that preoperative gut microbiota may not be a factor influencing susceptibility to SAE. However, the gut microbiota of the mice was markedly disrupted after the CLP operation (see Fig. S2A to C in the supplemental material). Compared with control mice, the richness and diversity of the intestinal microbiota were significantly decreased in SES mice, whereas SER mice had mildly dysregulated intestinal microbiota. Specifically, SES mice showed a lower Shannon index and a higher Simpson index than SER mice (Fig. 2A), suggesting a decrease in bacterial diversity in SES mice compared with that in SER mice. PCA data also showed distinctive microbial communities in the fecal microbiota between the SES and SER groups after operation (Fig. 2B and C). There was considerable interindividual variability in gut microbiota composition at the phylum level in mice that underwent an operation (Fig. 2D).

10.1128/msystems.01399-21.1 FIG S1 Variations in the composition of the gut microbiota in SES and SER mice before CLP. Fecal samples from mice were collected 1 day before CLP. (A) Composition of α-diversity (as assessed by the Shannon, Simpson, Chao, and Ace indices) in feces. (B) Scatter plots of PCA for gut microbiota composition showing the β-diversity of feces. (C) Relative abundance of gut microbiota at the phylum level. *n* = 6. Download FIG S1, EPS file, 1.6 MB. Copyright © 2022 Fang et al. 2022 Fang et al. https://creativecommons.org/licenses/by/4.0/ This content is distributed under the terms of the Creative Commons Attribution 4.0 International license.

10.1128/msystems.01399-21.2 FIG S2 Variations in the composition of the gut microbiota caused by sepsis in mice. Fecal samples from mice were collected 1 day before and 2 days after CLP. (A) Composition of α-diversity (as assessed by the Shannon, Simpson, Chao, and Ace indices) in feces. (B) Scatter plots of PCA for gut microbiota composition showing the β-diversity of feces. (C) Relative abundance of the gut microbiota at the phylum level. *n* = 12. *, *P* < 0.05; **, *P* < 0.01; ***, *P* < 0.001. Download FIG S2, EPS file, 1.7 MB. Copyright © 2022 Fang et al. 2022 Fang et al. https://creativecommons.org/licenses/by/4.0/ This content is distributed under the terms of the Creative Commons Attribution 4.0 International license.

We further analyzed the differences in gut microbiota composition between the SES and SER groups. At the family level, *Enterobacteriaceae* enrichment was observed in SES mice (Fig. 2E). Moreover, linear discriminant analysis effect size (LEfSe) revealed significant differences between the two groups. In particular, the order *Enterobacterales*, family *Enterobacteriaceae*, phylum *Proteobacteria*, and class *Gammaproteobacteria* were more enriched in the SES group, while the class *Bacteroidia*, phylum *Bacteroidota*, and order *Bacteroidales* were enriched in SER mice (Fig. 2F and G).

### Dependence of CLP-induced neuroinflammation on gut microbiota.

**FIG 3:**
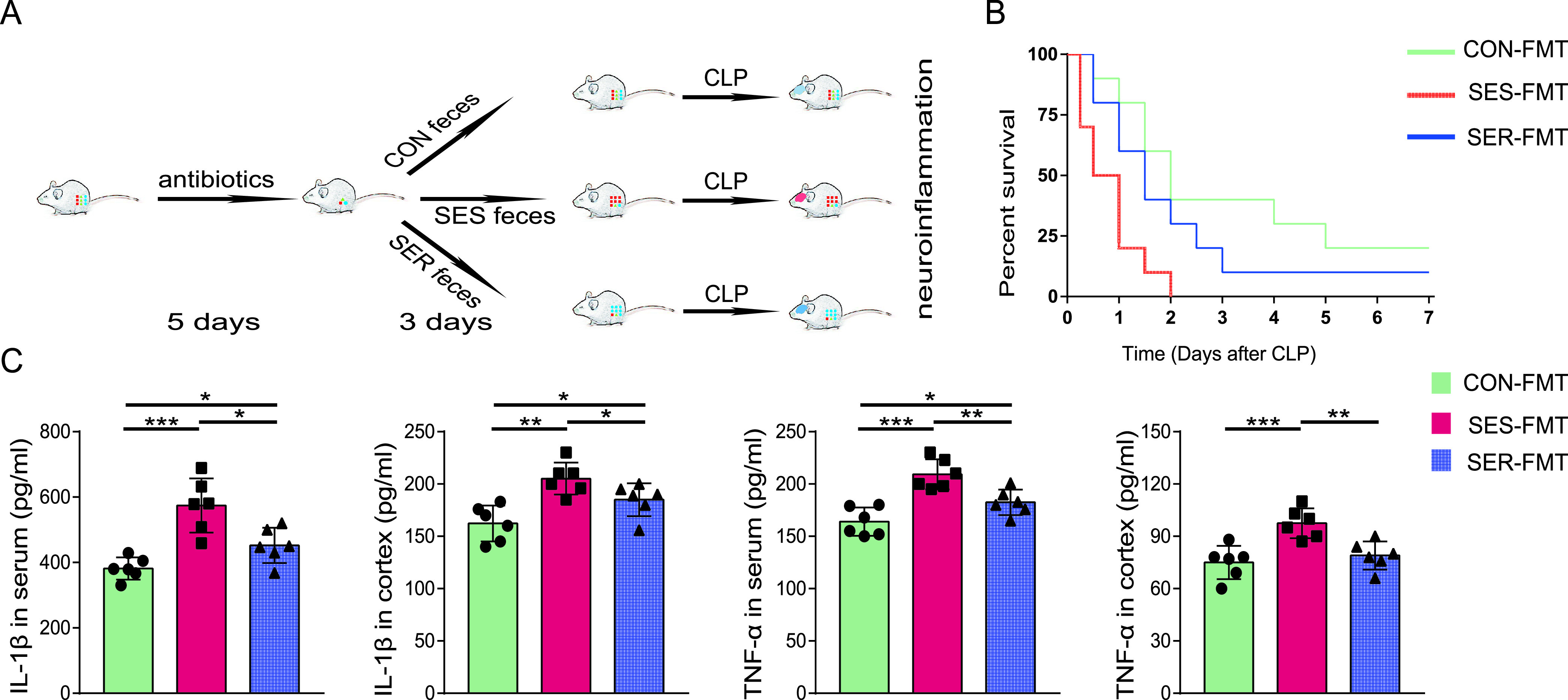
Dependence of CLP-induced neuroinflammation on the gut microbiota. (A) Mice were administered antibiotics once daily for 5 days and then transplanted with feces from healthy, SES, and SER mice once daily for 3 days. (B) Survival rate. (C) Levels of proinflammatory cytokines in serum and cortex of the recipient mice. *n* = 6 to 10. *, *P *< 0.05; **, *P *< 0.01; ***, *P *< 0.001.

After identifying significant differences between SES mice and SER mice with respect to postoperative gut microbiome composition, we sought to determine whether these differences contributed to the differential susceptibility to SAE. We performed an FMT experiment (Fig. 3A). After antibiotic treatment to deplete the intestinal microbiota for 5 days, mice received feces from healthy (CON-FMT), SES (SES-FMT), and SER (SER-FMT) mice (Fig. 3A). All mice that received feces from SES mice died within 48 h. The survival rate was 40% for mice receiving feces from healthy mice and 30% for mice receiving feces from SER mice (Fig. 3B). These results suggest that the gut microbiota of SER mice could provide a survival advantage after CLP compared to SES mice. In addition, mice in the CON-FMT group showed the lowest levels of proinflammatory cytokines in the serum and cortex among these three groups. In contrast to mice gavaged with feces from SER mice, mice that received feces from SES mice had significantly higher levels of IL-1β and TNF-α both in the serum and cerebral cortex (Fig. 3C). These data indicate that CLP-induced neuroinflammation could be transferrable by gut microbiota and that the gut microbiota from SER mice was a key factor in the resistance of mice to SAE.

### Enrichment of indole-3-propionic acid in feces from SER mice.

**FIG 4:**
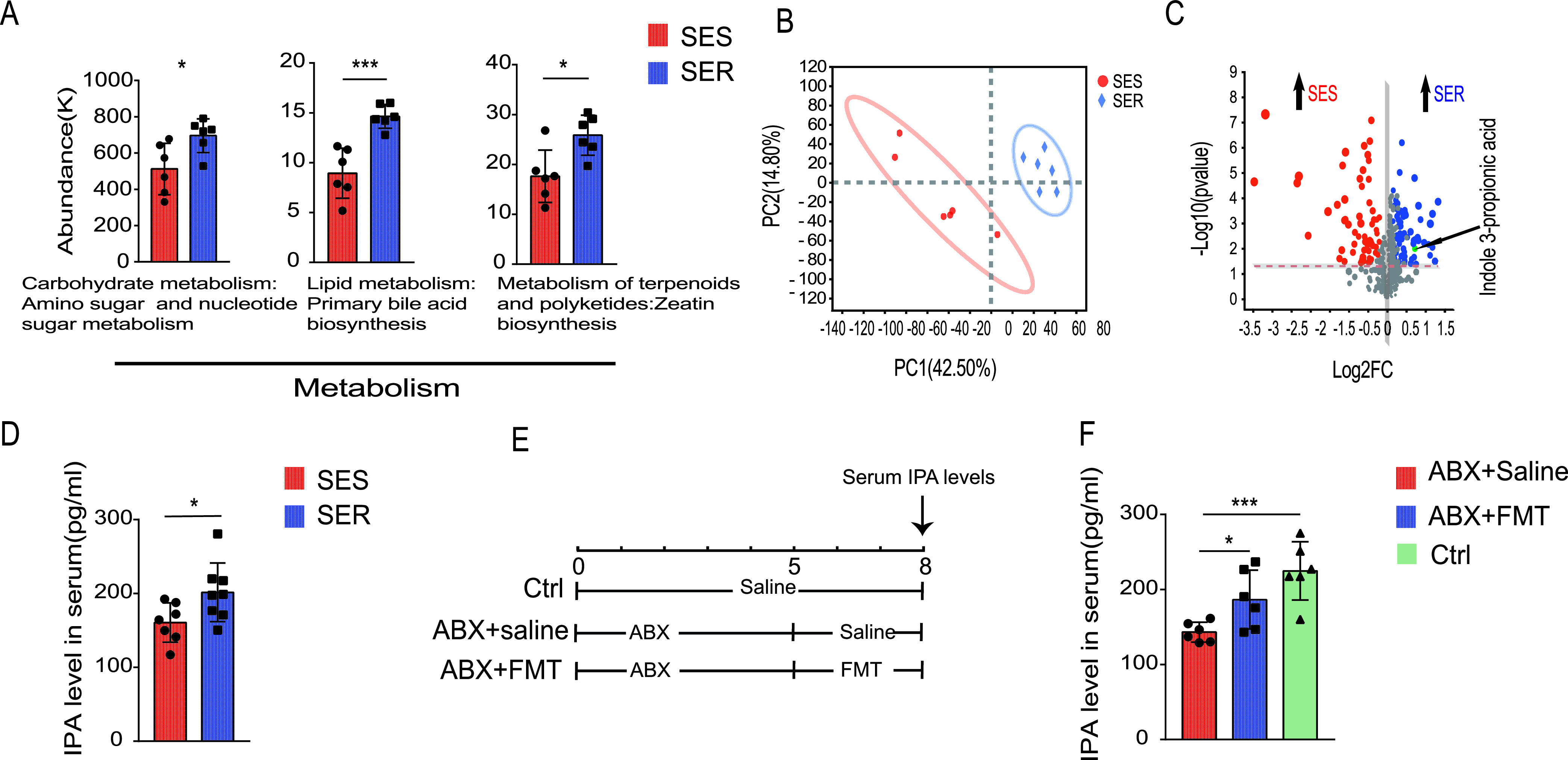
Gut microbiota of SES and SER mice after CLP showed distinct functions. Feces were collected from SER and SES mice on day 2 after the CLP operation. (A) Relative abundance of genes involved in the indicated pathways based on PICRUSt analysis. (B) Scatterplots of PCA for metabolomics in feces. (C) Volcano plot showing the results of metabolomics analysis. (D) IPA levels in serum from SER and SES mice. (E) Schematic diagram of the FMT experimental design and procedure. (F) Serum IPA levels. *n* = 6 to 7. *, *P *< 0.05; **, *P *< 0.01; ***, *P *< 0.001.

To explore the sepsis-induced functional differences of the gut microbiota between SES and SER mice, PICRUSt analysis, a computational approach to predict microbial physiological function at the genomic level, was conducted. Significant differences in the expression level of several genes associated with metabolism were observed. More specifically, the genomic abundance of certain pathways, such as the lipid metabolism, carbohydrate metabolism, terpenoid, and polyketide metabolism, was significantly diminished in SES feces compared to SER feces (Fig. 4A). Nontargeted metabolomics analysis of the gut microbiota was performed to further explore metabolic functions in SES and SER mice. PCA showed separation of the metabolite profiles of each group (Fig. 4B). Additionally, some metabolites were differentially enriched in the feces of the two groups (Fig. 4C). Indole-3-propionic acid (IPA) is a metabolite that was found to be neuroprotective in previous studies (11). IPA was significantly enriched in the feces of SER mice, implying that it may also play a protective role in the pathogenesis of SAE (Fig. 4C). Moreover, the serum levels of IPA in SER mice were higher than those in SES mice (Fig. 4D).

Since serum IPA concentrations differed between SER and SES mice, we wondered whether IPA concentrations in mouse serum were affected by the intestinal microbiota. Mice were administered antibiotics (ABX) for 5 days to deplete their intestinal microbiota, followed by gavage with feces from healthy mice for 3 days to restore their intestinal microbiota (Fig. 4E). The results showed that the IPA levels in serum decreased significantly after ABX treatment, but the IPA levels partially reverted after administration of feces from healthy mice (Fig. 3F). Our data clearly demonstrate that changes in the gut microbiota affect serum IPA levels.

### Indole-3-propionic acid protected mice against CLP-induced SAE and death.

**FIG 5:**
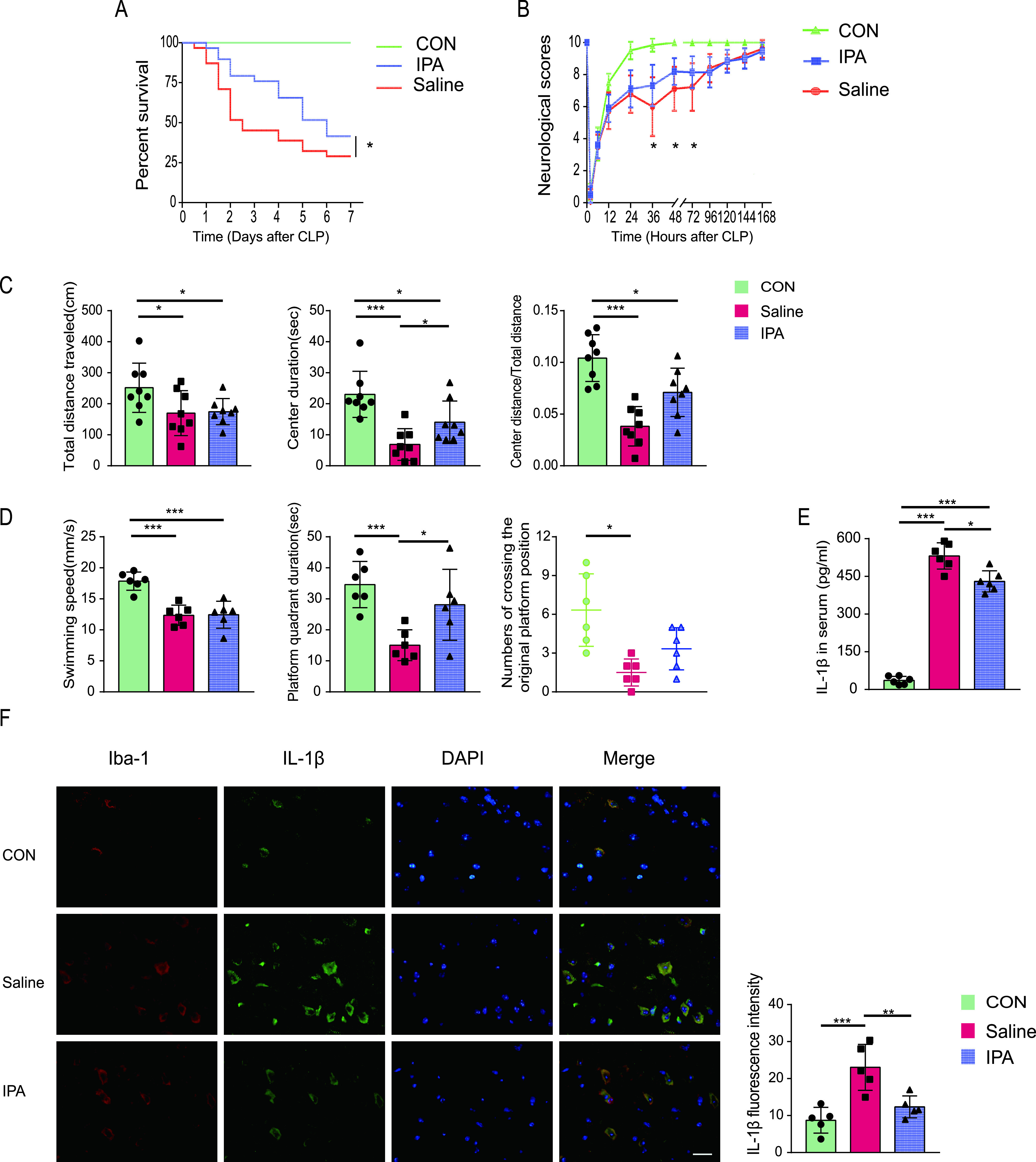
IPA showed a protective effect against CLP-induced death and SAE in mice. (A) Survival rate. (B) Neurological scores. (C) Results of the open field test performed for SES and SER mice on day 2 after CLP. Total distance traveled, center duration, and ratio of center distance to total distance are shown. (D) Results of the Morris water maze test, which was performed beginning on day 7 after CLP. The number of times of the mice crossed the original platform position, swimming speed, and original platform quadrant duration on the day of probe trials are shown. (E) Serum IL-1β levels. (F) Representative image of IL-1β (green) and Iba-1 (red) immunofluorescence staining in the cortex of mice. Scale bar, 20 μm. *n* = 5 to 20. *, *P* < 0.05; **, *P *< 0.01; ***, *P* < 0.001.

Next, we treated mice with IPA or saline to assess the protective effect of IPA against CLP-induced SAE and death. Survival analysis showed better survival for IPA-treated mice than saline-treated mice (Fig. 5A). Additionally, neurologic assessment showed greater activity in the neurological reflexes of IPA-treated mice at 36, 48, and 72 h compared to that of saline-treated mice subjected to CLP (Fig. 5B). Open field tests showed a significant increase in the duration spent in the center and the ratio of the center distance to the total distance of IPA-treated mice compared with that of saline-treated mice. However, no significant between-group difference was observed with respect to the total distance traveled. These findings suggested that IPA reduced anxiety but did not improve mobility in CLP-operated mice (Fig. 5C). The Morris water maze test showed that the frequency of crossing the original platform location and duration spent in the platform quadrant during the probe trial on the sixth day in the IPA-treated group were markedly increased compared with those of the saline-treated group. However, there was no significant difference in the swimming speeds (Fig. 5D). These data indicate that IPA may have restored the CLP-induced cognitive impairment in the CLP model. In addition, IPA-treated septic mice had lower levels of IL-1β in the serum and cortex than saline-treated mice (Fig. 5E and F). There was a high degree of overlap between IL-1β-positive cells and microglia (Fig. 5F), suggesting the involvement of microglia in the pathophysiology of SAE development.

### Indole-3-propionic acid inhibited the activation of the NLRP3 inflammasome in lipopolysaccharide-stimulated microglia.

**FIG 6:**
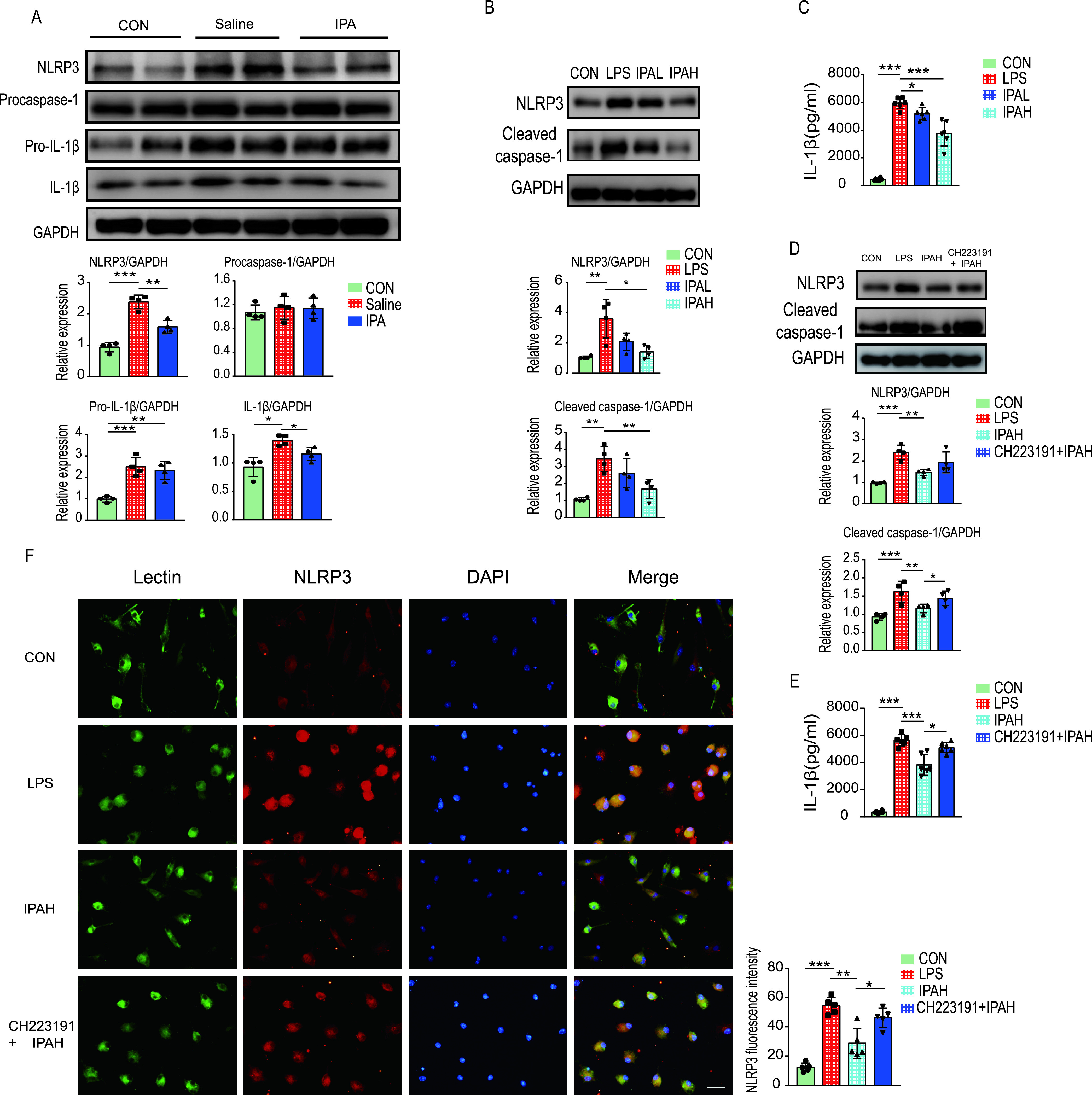
Indole-3-propionic acid inhibits the NLRP3 inflammasome. (A) Mice were pretreated with IPA or saline for 5 days and then underwent CLP or sham operation. The cerebral cortex was collected at 36 h after CLP in mice. The expression levels of NLRP3, procaspase-1, pro-IL-1β, IL-1β, and glyceraldehyde-3-phosphate dehydrogenase (GAPDH) in the cortex were detected by Western blotting and quantified. (B, C) Mouse primary microglia were pretreated with different concentrations of IPA (IPAL, 0.5 mM; IPAH, 1.0 mM) for 1 h and stimulated with LPS for 12 h. (B) The expression levels of NLRP3 and cleaved caspase-1 in cell extracts were detected by Western blotting and quantified. (C) The IL-1β levels in the supernatant were detected by enzyme-linked immunosorbent assay (ELISA). (D, E, F) Mouse primary microglia were pretreated with CH223191 (10 μM) for 1 h. This was followed by treatment with IPA (IPAH, 1.0 mM) for 1 h followed by LPS stimulation for 12 h. (D) The expression levels of NLRP3 and cleaved caspase-1 in cell extracts were detected by Western blotting and quantified. (E) The IL-1β levels in the supernatant were detected by ELISA. (F) Representative image of NLRP3 (red) and lectin (green) immunofluorescence staining in primary microglia. Scale bar, 20 μm. *n* = 4 to 5. *, *P *< 0.05; **, *P *< 0.01; ***, *P *< 0.001.

Based on the results of immunofluorescent staining, we speculated that the levels of IL-1β in the cortex of CLP mice may be reduced by IPA and that microglia were the main effector cells involved in this pathophysiology. Therefore, we wondered whether IPA suppresses cortical inflammation by inhibiting the activation of the NLRP3 inflammasome in microglial cells. The NLRP3 inflammasome has been reported to be a key player in the development of SAE (19), but the effect of IPA on the NLRP3 inflammasome is unclear. Western blotting analyses showed an apparent increase in the levels of NLRP3, pro-IL-1β, and IL-1β in the cortex of CLP-operated mice compared with those of sham-operated mice. These findings suggest a critical role of the NLRP3 inflammasome in SAE. Treatment with IPA resulted in a decrease in the levels of NLRP3 and IL-1β in the cortex compared with those in CLP mice treated with saline. In addition, mice in the IPA and saline groups showed similar levels of procaspase-1 and pro-IL-1β in the cortex (Fig. 6A). These results suggest that IPA could inhibit NLRP3 expression and IL-1β production.

Sufficient NLRP3 protein expression is essential for NLRP3 inflammasome formation and activation (20). Given that IPA negatively regulates NLRP3 expression, we tested whether IPA could inhibit NLRP3 inflammasome activation *in vitro*. NLRP3 activation promotes the cleavage of procaspase-1, and then mature caspase-1 promotes the cleavage of pro-IL-1β to IL-1β (20). Primary microglia were pretreated with IPA for 1 h, followed by stimulation with lipopolysaccharide (LPS) for 12 h. After stimulation with LPS, the levels of NLRP3, cleaved caspase-1, and IL-1β in cells showed a significant increase compared with controls. However, the increase was markedly restored after an LPS challenge in the presence of IPA (1 mM) (Fig. 6B and C). These data indicate that IPA inhibits NLRP3 inflammasome activation and the subsequent IL-1β secretion.

The aryl hydrocarbon receptor (AhR) has been shown to negatively regulate NLRP3 inflammasome activity (21). AhR, a ligand-dependent transcription factor, can be activated by diverse ligands including indole-based compounds (22, 23). Thus, we hypothesized that IPA inhibited the activation of the NLRP3 inflammasome, and this inhibition could be reversed by an AhR antagonist. As shown in Fig. 6D to F, pretreatment with an AhR antagonist (CH223191) attenuated the effects of IPA on the inhibition of NLRP3 expression, caspase-1 cleavage, and IL-1β secretion in the primary microglial cells after an LPS challenge *in vitro*. These results suggest that the inhibitory effect of IPA on NLRP3 inflammasome activation and IL-1β secretion in microglia after LPS challenge might be mediated by AhR.

## DISCUSSION

In this study, we demonstrated that the variability in sepsis-induced gut dysbiosis mediates differential susceptibility to SAE in CLP-induced experimental sepsis mice. The gut microbiota from SER mice enriched a neuroprotective metabolite, IPA, that appeared to protect mice from SAE. The potential underlying mechanism of the protective effect of IPA may be mediated via the inhibition of NLRP3 inflammasome activation and IL-1β secretion in microglia. These anti-inflammatory effects of IPA may be mediated by AhR. These results enhance our understanding of the role of intestinal microbiota in sepsis. In particular, gut microbiota-derived IPA may serve as a potential therapeutic agent to prevent neuroinflammation in SAE.

In previous studies, FMT, probiotics, or certain drugs were shown to alleviate SAE by affecting the intestinal microbiota (18, 24). These studies indicated that the gut microbiota may be an upstream regulator of SAE, which is consistent with our findings. However, these studies did not explore interindividual variability in SAE occurrence. In this study, a mouse model of sepsis was established using the CLP method, and we observed individual differences in neural reflexes and mobility among the model mice. Combining the results of neurological scores, behavioral tests, and IL-1β levels in the cerebral cortex, a neurological score of ≤6 at 36 h postoperatively was found to be a rapid method for screening SAE-susceptible model mice. This screening method may provide a methodological reference for future studies related to SAE.

Induction of gut dysbiosis in septic patients has been previously reported (25, 26). We found that the feces of SES mice were enriched with *Enterobacteriaceae*. *Enterobacteriaceae* overgrowth in the gut could exacerbate brain infarction and systemic inflammation in stroke patients (27). Moreover, *Enterobacteriaceae* expansion in the gut significantly increases the risk of bloodstream invasion, sepsis, and death (28). In the present study, gut *Enterobacteriaceae* expansion may exacerbate systemic inflammation and bacterial dissemination. The FMT experiment demonstrated that the gut microbiota was an upstream mediator of brain inflammation during sepsis and that sepsis-induced differences in the gut microbiota were an important factor for individual differences in response to SAE. Of note, IPA, a molecule with proven neuroprotective properties (29), was enriched in feces from SAE-resistant mice.

Previous studies have demonstrated that IPA can cross the blood-brain barrier (30) and modulate astrocyte activation and neuroinflammation; it can also inhibit neuronal death induced by endoplasmic reticulum stress (31). In addition, supplementation with the microbial metabolite IPA was shown to reduce neuroinflammation in mice with encephalomyelitis (29). These studies indicate the protective effect of IPA against neuronal inflammation, which prompted us to select IPA from the metabolites enriched in SER feces as a possible protective substance against SAE. Consistent with previous findings (29, 32), we also observed a beneficial role for IPA in SAE, which was related to its anti-inflammatory effects. In the present study, the direct effect of IPA on microglia may be responsible for the reduction in neuroinflammation after IPA treatment. In addition, less systemic inflammation after IPA treatment may be another reason for the decreased neuroinflammation that was observed compared with the saline treatment group.

Microglia are important to the development of SAE (33, 34). Activated microglia overexpress IL-1β, which can cause excitatory synaptic damage that leads to cognitive impairment in sepsis (35). IL-1β secretion is regulated by NLRP3, which is a crucial pattern recognition receptor. Activation of NLRP3 promotes procaspase-1 cleavage and IL-1 secretion upon the recognition of pathogen-associated molecular patterns (PAMPs) or danger-associated molecular patterns (DAMPs). Reducing IL-1β levels through inhibition of NLRP3 in microglia was shown to improve cognitive function in sepsis (19, 36). Consistently, we found that IPA inhibited LPS-mediated NLRP3 inflammasome activation and IL-1β secretion in microglia. This finding suggests that IPA modulates inflammatory responses in sepsis, at least in part, by inhibiting the activation of the NLRP3 inflammasome. In addition, the reduction in systemic inflammation in IPA-treated mice caused a reduction in neuroinflammation, resulting in a decrease in DAMPs in the brain, which may be another reason for the decrease in NLRP3 activation.

Recent studies suggest that IPA may control microglia-activated inflammation through a mechanism mediated by AhR (29, 37). In addition, AhR was shown to be a negative regulator of NLRP3 inflammasome activity at the transcriptional level (21). Thus, activation of AhR and inhibition of the NLRP3 inflammasome is a plausible strategy to alleviate neuroinflammation in microglia. In the present study, the inhibitory effect of IPA on the NLRP3/IL-1β axis was abolished by an AhR antagonist *in vitro*, suggesting that IPA exerts its anti-inflammatory effect possibly via AhR in microglia.

Some limitations must be acknowledged in this study. First, since IPA is a gut microbiota-derived metabolite, no antibiotics were used in the IPA delivery experiments to eliminate the possible effects of additional IPA produced by the microbiota. Second, whether IPA reduces bacteremia and thus protects mice against SAE is unclear. Third, AhR-knockout mice were not used to confirm that IPA affects mouse survival via AhR in microglia.

### Conclusions.

In summary, by associating a microbial metabolite, IPA, as a neuroprotective compound, our study suggests that the gut microbiota is an upstream regulator of SAE and that the variability in sepsis-induced gut dysbiosis mediates the differential susceptibility to SAE in CLP-induced experimental sepsis mice. Our findings reveal potential mechanisms by which the gut microbiota mediates SAE susceptibility and enrich our understanding of the “microbiota-gut-brain” axis during sepsis progression.

## MATERIALS AND METHODS

### Animal model.

Specific pathogen-free male C57BL/6 mice (age, 6 to 8 weeks) were used in this study. All experimental animal studies were approved by the local Animal Care and Use Committee of Guangdong Provincial People's Hospital. A mouse model of sepsis was induced by performing CLP (9). Briefly, mice were anesthetized with pentobarbital. After skin preparation and disinfection of the abdomen, a midline incision of approximately 1 cm was made to expose the cecum. The middle of the cecum was ligated and a single through-and-through puncture was performed with a 21-gauge needle between the ligation site and end of the cecum. After extruding a small amount of fecal material through the puncture, the cecum was gently repositioned in the abdominal cavity and the incision was sutured. Sham-treated mice underwent laparotomy and manipulation to expose the cecum without ligation and puncture. All mice were resuscitated by subcutaneous injection of 1 mL of saline. Before modeling sepsis, mice in the indole-3-propionic acid (IPA) (Sigma; 220027) treatment group received IPA by gavage (25 mg/kg of body weight dissolved in saline) once daily for 5 days and once within 1 day after modeling (modified treatment protocol based on previous studies [29, 38] and our pilot trials). Equal amounts of saline were administered to mice in the control group. All mice were provided *ad libitum* access to feed and water and housed in a controlled environment. Mouse feces were collected 1 day before and 2 days after the CLP operation and stored at −80°C. Some mice were sacrificed after 36 h to harvest tissues.

### Fecal microbiota transplantation.

FMT was performed according to the modified method described elsewhere (9). Briefly, male C57BL/6 mice (age, 6 to 8 weeks) received feces from donor mice (control, susceptible, and resistant) after depletion of the gut microbiota by antibiotic treatment. The antibiotic regimen that was administered consisted of 100 mg/kg vancomycin, 200 mg/kg neomycin sulfate, 200 mg/kg metronidazole, and 200 mg/kg ampicillin. The regimen was administered by gavage once daily for 5 days. The fecal administration regimen consisted of resuspending donor feces in saline at a concentration of 0.125 g/mL and then administering 0.15 mL of this suspension to mice by oral gavage once daily for 3 days. After 3 days, mice were subjected to CLP and some were euthanized 12 h later to harvest tissues.

### Primary culture of microglia and treatment.

Pure primary microglia cultures were obtained from the cortex of 1-day-old C57BL/6 mice according to a modified method described elsewhere (39). In brief, mouse cortices obtained by dissection under aseptic conditions were cut into pieces and digested with trypsin (final concentration, 0.125%) at 37°C for 10 min. After the addition of fetal bovine serum (FBS) to stop the reaction, the suspension was filtered through a 200 mesh filter and then centrifuged at 1,000 rpm for 5 min. Cells were resuspended in Dulbecco modified Eagle medium (DMEM)/F-12 (10% FBS, 1% penicillin/streptomycin), seeded in several flasks precoated with poly-l-lysine (PLL) (0.1 mg/mL), and grown at 37°C with 5% CO_2_ for 12 to 14 days. Small amounts of medium were added every 3 days thereafter, and the growth of the cells was observed under a microscope. Culture flasks were shaken for 1 h (200 rpm, 37°C) to detach microglial cells, and the medium containing microglia was collected for centrifugation (1,000 rpm, 5 min). The supernatant was removed, and the obtained pure microglia were resuspended in DMEM/F-12 (10% FBS, 1% penicillin/streptomycin) and seeded onto subcultures precoated with PLL. After 24 h of pure microglial culture, the cells were treated with IPA (0.5 mM, IPAL group; 1.0 mM, IPAH group). Then, they were dissolved in dimethyl sulfoxide (DMSO) for 1 h followed by LPS (1 μg/mL) stimulation for 6 h. Cells in the LPS group were stimulated with LPS only, and cells in the control group were not stimulated. Cells in the CH223191+IPAH group were pretreated with CH223191 (10 μM, dissolved in DMSO) for 1 h. Then, they were treated with IPA (1.0 mM) for 1 h followed by LPS (1 μg/mL) stimulation for 6 h.

Other materials and methods are described in Text S1 in the supplemental material.

10.1128/msystems.01399-21.3 TEXT S1 Supplemental materials and methods. Download Text S1, DOCX file, 0.02 MB. Copyright © 2022 Fang et al. 2022 Fang et al. https://creativecommons.org/licenses/by/4.0/ This content is distributed under the terms of the Creative Commons Attribution 4.0 International license.

### Statistical analyses.

Data are presented as the mean ± standard deviation. Statistical analysis was conducted with either Student's *t* test or analysis of variance (ANOVA). Post-hoc analysis was performed for the ANOVA testing. *P* values of <0.05 were considered indicative of statistical significance.

### Data availability.

The data sets used and analyzed during the current study are available from the authors upon reasonable request, and some have already been included in the paper. Raw sequence data of the microbiota that support the findings in our study have been deposited into NCBI’s Sequence Read Archive under accession number PRJNA779842.
