# Supplementary material for: Sepsis-Induced Gut Dysbiosis Mediates the Susceptibility to Sepsis-Associated Encephalopathy in Mice
Source: mSystems. 2022 Jun 1;7(3):e01399-21. doi: 10.1128/msystems.01399-21 (PMC9239149; doi:10.1128/msystems.01399-21)
Supplement: TEXT S1 [file msystems.01399-21-s0003.docx]

**Neurological scoring**

Neurological function of mice was measured as described in previous reports(1), focusing on the pinna reflex, corneal reflex, righting reflex, tail flexion reflex, and escape response. The pinna reflex test was assessed by touching the auricle to elicit a head shake; the corneal reflex test was evaluated by lightly touching the cornea with a cotton swab to cause blinking or head shaking; the righting reflex test was assessed by placing the mice in a supine position and observing whether they could return to a spontaneous upright position. The tail flexion reflex and escape response were tested by briefly pinching the tail to elicit a withdrawal response or head turn to avoid the injurious stimulus. Absence of reflex was scored as 0, weak reflex was scored as 1, and normal reflex was scored as 2. The maximum score for each mouse was 10.

**Open field test**

Mice who survived 36 hours after CLP surgery were subjected to open field test to assess their anxiety behavior and motor ability. Each mouse was gently placed at the center of a square area (60×60×60 cm) and then allowed to explore for 5 min. The trajectory of mouse activities was recorded using a video tracking system. After each test, the field was cleaned with 75% alcohol to remove odor disturbance.

**Morris water maze test**

Morris water maze test was performed on surviving septic mice 7 days after CLP surgery to assess spatial learning memory as previously described(2). The test was conducted in a circular pool (diameter: approximately 1.2 m) of water (25.0±1.0 °C) with a depth of 25 cm. Graphic markings were made in the visual range of the mice. The pool was divided into four quadrants, one of which had a raised platform (diameter: 9 cm) 1.0 cm below the water surface. On the first day, mice were placed in the water and allowed to swim freely for 120 seconds in order to acclimatize them to the circumstances. On the subsequent 4 days, mice were randomly placed in water facing the wall of the pool in one of the quadrants, and each mouse entered the water once in each quadrant. The trajectory and time of movement of mice were recorded. If the mice failed to locate the platform within 120 s, they would be placed on the platform for an additional 60 s. On the sixth day of probe trials, the platform was removed. Mice were released in the water from the same position and allowed to swim for 120s. The number of times they crossed the site of the original platform, the time spent in the original platform quadrant, and their swimming speed were recorded.

**Fecal microbiota composition analysis**

Microbial DNA was extracted from fecal samples using the Mag-Bind soil DNA kit (Omega Bio-Tek, USA) according to the manufacturer’s protocols. PCR amplification of the 16S rRNA gene was performed on the V3-V4 hypervariable region as previously reported. Sequencing was conducted using Illumina's Miseq PE300 platform (Illumina, San Diego, CA, USA) by Majorbio (Shanghai, China). Using UPARSE software (version 7.1), the sequences with clustering of operational taxonomic units (OTUs) and chimeras were removed at a similarity level of 97%. Bioinformatics analyses were performed using Mothur and QIIME2.0 software. Alpha diversity was evaluated using Shannon, Simpson, Chao, and ACE indices, and the beta diversity was analyzed using principal coordinate analysis based on Bray-Curtis distance. Microbiota taxonomy was used to assess specific community differences between groups, and the linear discriminant analysis effect size (LEfSe) Galaxy module was used for additional analyses examining biologic consistency and effect relevance. Phylogenetic Investigation of Communities by Reconstruction of Unobserved States (PICRUSt) software was utilized to combine the 16S amplicon sequences with Kyoto Encyclopedia of Genes and Genomes for gene function prediction.

**Metabolomics analysis**

Liquid chromatography -mass spectrometry was performed on an AB Sciex TripleTOF 5600TM mass spectrometry system (AB SCIEX, USA) for nontargeted metabolomics analysis, as described elsewhere. The raw data were further analyzed using the metabolomics software Progenesis QI (Waters Corporation, Milford, USA) for peak detection and alignment, and a data matrix was generated for bioinformatics analysis. Principal component analysis (PCA) using unsupervised methods was performed to demonstrate repeatability within data groups and variability between groups.

**Western blot analysis**

Proteins were extracted from mice cerebral cortex or cells using a protein extraction kit (Best Bio, BB-3101-100T), according to the manufacturer’s instructions. The protein concentrations were determined using BCA kit (Thermo Scientific). After protein denaturation by heating (99 °C, 10 min), samples of supernatants containing 40 µg of cortical protein or 25 µg of cellular protein were separated by 12% SDS-polyacrylamide gel electrophoresis, transferred onto polyvinylidene difluoride membranes for immunoblot analysis, and sealed with 5% skim milk for 1 hour at room temperature. Membranes were incubated overnight at 4 °C with the following primary antibodies: IL-1β (1:1000, Abcam, ab200478), NLRP3 (1:1000, Abcam, ab263899), caspase-1 p20 (1:500, Affinity, AF4005), and GAPDH (1:1000, Abcam, ab181602). After washing off the free primary antibody, the membranes were incubated with the secondary antibody (1:5000, Cell Signaling Technology, 7074S) for 1 h at room temperature. Protein bands were detected by chemiluminescence kit (Millipore, WBKLS0500) and visualized using Image Quant LAS500 (GE Healthcare Bio-Sciences AB). The intensity of the protein bands was quantified using Image J software. Protein expressions were normalized to those of GAPDH.

**Enzyme-linked immunosorbent assay**

Cerebral cortex protein extracts, serum, and cell supernatants were collected. The levels of TNF-α and IL-1β were measured by enzyme-linked immunosorbent assay (ELISA) kit (Cusabio), according to the manufacturer's instructions. Serum levels of IPA were measured by ELISA kit (Boshen biotechnology). The levels of inflammatory factors in the tissues were corrected accordingly to the total protein concentration in the extracts.

**Immunohistochemistry**

Immunofluorescence assays were performed on both brain tissue and primary microglia cells. For tissue immunofluorescence staining, frozen sections of brain tissues were blocked by blocking buffer (containing 0.1% Triton x-100, 1% BSA, 10% fetal bovine serum, prepared in PBS) for 1 hour at room temperature, and then incubated overnight in the primary antibody solution (IL-1β 1:500, Affinity, AF5103; Iba-1 1:400, GeneTex, GT10312) at 4 °C. After washing, the sections were incubated with fluorescent anti-rabbit (1:300, Abcam, ab150077) or anti-mouse secondary antibodies (1:300, Abcam, ab150116) for 1 hour at room temperature. Nuclear staining was performed using DAPI (Sigma, D9542). For cellular immunofluorescence staining, cells were seeded in 6-well plates with one coverslip placed in advance. The cells were fixed with 4% paraformaldehyde for 8 min at room temperature and blocked using the blocking buffer. After washing, the cells were incubated overnight with anti-NLRP3 primary antibody (1:300, Affinity, DF7438) at 4 °C and then exposed to fluorescent anti-rabbit secondary antibody (1:300, Abcam, ab150080) and lectin (1:300, Sigma, SLBX1079) for 1 hour at room temperature. Finally, the coverslips were obtained and stained with DAPI. Fluorescence images were acquired at 40× using a fluorescence microscope.

**Statistical analysis**

The results are expressed as the mean ± standard deviation. Two-tailed Student’s t-test was used for statistical evaluation. Statistical differences between groups were analyzed using a significance level set at p<0.05.

**REFERENCES**

1. Kafa IM, Bakirci S, Uysal M, Kurt MA. 2010. Alterations in the brain electrical activity in a rat model of sepsis-associated encephalopathy. Brain Res 1354:217-26.

2. Zhou Q, Lin L, Li H, Wang H, Jiang S, Huang P, Lin Q, Chen X, Deng Y. 2021. Melatonin Reduces Neuroinflammation and Improves Axonal Hypomyelination by Modulating M1/M2 Microglia Polarization via JAK2-STAT3-Telomerase Pathway in Postnatal Rats Exposed to Lipopolysaccharide. Mol Neurobiol 58:6552-6576.
